# O13 Back to the Future: attempting to distinguish between inflammatory and non-inflammatory back pain

**DOI:** 10.1093/rap/rkaa054.001

**Published:** 2020-11-03

**Authors:** James Brighouse, Nina Mossop, Milly Munn, Robert Schneider, Kathryn Shepherd, Nick Wilkinson, Vinay Shivamurthy

**Affiliations:** Rheumatology, Evelina London Children's Hospital, United Kingdom

## Abstract

**Case report - Introduction:**

Back pain is highly prevalent, affecting 40% of the paediatric population, with non-inflammatory causes accounting for the majority cases. However, the presentation of inflammatory back pain can be non-specific and a high degree of suspicion is necessary, particularly with the presence of risk factors such as HLA B27 positivity and personal and family history of psoriasis, inflammatory bowel disease, and acute uveitis.

**Case report - Case description:**

A previously well 14-year-old Caucasian girl was referred due to persistent lower back pain, HLA B27 positivity, and a history of acute uveitis. She reports that her pain began around the time of a road traffic accident two years previously. The pain was persistent but varied in intensity, worsened throughout the day, on bending forwards, and during bumpy car journeys, and on occasion was mildly relieved by ibuprofen. She later also developed neck pain and right lateral thigh pain. Despite her pain she was able to continue walking and swimming but found that she had to stop trampolining and cycling. She had recently been treated for a painful, red eye with topical treatment.

On examination she had full range of movement of her spine and tenderness over her sacroiliac joints and plantar fascia insertion points, as well as more widespread muscle tenderness. Her bloods demonstrated normal inflammatory markers and MRI of her lumbar spine, pelvis, and sacroiliac joints showed a subtle disc degeneration with bulge at L5/S1 without nerve root compression or evidence of inflammation.

An MDT approach was taken to manage her pain, with input from occupational therapy, discussing pain processing, pacing, pain management strategies, and sleep hygiene, and physiotherapy including hydrotherapy and a home exercise programme. Despite these interventions, her pain progressed and resulted in further functional impairment. Following MDT discussion, an MRI, already repeated after her first appointment, was performed for the third time which on this occasion demonstrated bilateral sacroiliitis with subchondral sclerosis, erosions, and bone marrow oedema. A diagnosis of enthesitis-related arthritis was made and treatment with diclofenac and etanercept was initiated.

**Case report - Discussion:**

With HLA B27 positivity and a history of acute uveitis, she was clearly at risk of developing inflammatory arthritis, but without evidence of inflammation on imaging or bloods, treatment with immunomodulatory drugs was not indicated. With a high prevalence of non-inflammatory musculoskeletal pain in adolescent females and the significant rate of persistent chronic pain into adulthood for untreated patients, early MDT input in this patient’s management was essential. While management of non-inflammatory pain is primarily driven by therapists, this case highlights the benefit of communication within the MDT and the important role of the medical team, in regularly reviewing the diagnosis, particularly where symptoms evolve or do not follow the expected clinical course.

**Case report - Key learning points:**

In the presence of risk factors for spondyloarthropathy, a diagnosis of non-inflammatory back pain needs to be regularly reviewed and evolving symptoms, clinical deterioration, and patient or parent concerns need to be addressed. Although important, HLA B27 positivity has an overall penetrance of less than 20% and therefore does not exclude a diagnosis of non-inflammatory back pain. However, when coupled with history of uveitis or significant family history, repeat imaging should be considered.

